# Mucoadhesive Budesonide Solution for the Treatment of Pediatric Eosinophilic Esophagitis

**DOI:** 10.3390/ph17050550

**Published:** 2024-04-24

**Authors:** Antonio Spennacchio, Antonio Lopalco, Giuseppe Francesco Racaniello, Annalisa Cutrignelli, Flavia Maria la Forgia, Sergio Fontana, Fernanda Cristofori, Ruggiero Francavilla, Angela Assunta Lopedota, Nunzio Denora

**Affiliations:** 1 Department of Pharmacy-Pharmaceutical Sciences, University of Bari Aldo Moro, 70125 Bari, Italy; antonio.spennacchio@uniba.it (A.S.); antonio.lopalco@uniba.it (A.L.); giuseppe.racaniello@uniba.it (G.F.R.); annalisa.cutrignelli@uniba.it (A.C.); angelaassunta.lopedota@uniba.it (A.A.L.); 2 Centro Studi e Ricerche “Dr. S. Fontana 1900–1982”, Farmalabor s.r.l., 76012 Canosa di Puglia, Italy; f.laforgia@farmalabor.it (F.M.l.F.); s.fontana@farmalabor.it (S.F.); 3 Interdisciplinary Department of Medicine, Paediatric Section, University of Bari Aldo Moro, Paediatric Hospital Giovanni XXIII, 70125 Bari, Italy; fernandacristofori@gmail.com (F.C.); ruggiero.francavilla@uniba.it (R.F.)

**Keywords:** budesonide, eosinophilic esophagitis, mucoadhesion, pediatric medicine, compounded formulation

## Abstract

Eosinophilic Esophagitis is an antigen-mediated inflammatory disease characterized by thickening of the esophageal wall, leading to dysphagia, vomiting, reflux, and abdominal pain. This disease can be treated with a therapeutic approach ranging from diet to pharmacological therapy. Jorveza^®^ (budesonide) and Dupixent^®^ (dupilumab) are treatments for Eosinophilic Esophagitis approved by the European Medicines Agency in adults but not in children. Budesonide-based extemporaneous oral liquid suspensions could be prepared for pediatric use. The main limit of this formulation is that budesonide needs a longer residence time on the esophageal mucosa to solubilize and diffuse in it to exert its local anti-inflammatory effect. Herein, we propose the development of an extemporaneous mucoadhesive oral budesonide solution for the pediatric population. A liquid vehicle containing hydroxypropyl-beta-cyclodextrin as a complexing agent and carboxymethylcellulose sodium as a mucoadhesive excipient was used to prepare budesonide-based formulations. A stable solution at a concentration of 0.7 mg/mL was successfully prepared and characterized. The formulation showed rheological and mucoadhesive properties suitable for an Eosinophilic Esophagitis local prolonged treatment. In this way, pharmacists can prepare stable budesonide-based mucoadhesive solutions, providing both patients and physicians with a new therapeutic option for Eosinophilic Esophagitis pediatric treatment.

## 1. Introduction

Eosinophilic Esophagitis (EoE) is an antigen-mediated esophageal inflammatory disease. EoE diagnosis is based on esophageal biopsy that highlights an eosinophil-predominant inflammation with a peak eosinophil count of ≥15 per high-power field (eos/hpf) [1,2]. The condition is characterized by the elevated presence of eosinophilic white blood cells and results in a thickening of the esophageal wall leading to dysphagia, vomiting, reflux, and abdominal pain [1,3,4]. Moreover, EoE causes the remodeling of the epithelial cells with a loss of functionality, causing the described symptoms [5,6]. Even if the prevalence of EoE is increasing in the EU, the disease is still rare with prevalence rates significantly higher in adults than children [7]. This disease can be treated with a therapeutic approach ranging from the elimination of harmful foods from the diet and avoiding specific allergens to endoscopy therapy and pharmacological therapy [8,9]. Unfortunately, even though it is often a safe and effective practice, a restrictive diet is characterized by low compliance. Moreover, it can lead to weight loss, and it is also economically unsustainable for long-term treatments [10]. For this reason, pharmacological therapies are required. Proton Pump Inhibitors could be considered a safe and effective initial therapeutical option, but not all patients respond to these active pharmaceutical ingredients (APIs) [11,12,13]. Jorveza^®^ (budesonide—BU) and Dupixent^®^ (dupilumab) are approved treatments for EoE by the European Medicines Agency (EMA) in adults but not in children. In this context, off-label topical corticosteroids, such as BU and fluticasone, are often proposed as a valuable therapeutic strategy [14,15,16]. BU is a second-generation glucocorticoid that has good local anti-inflammatory action and reduced systemic side effects because of extensive first-pass hepatic metabolism [17,18,19]. Therefore, BU use for EoE is steadily increasing, and it is becoming a drug of choice for the treatment of this disease. However, it is classified as a class II drug in the Biopharmaceutical Classification System (BCS) because of its specific physicochemical characteristics, such as poor solubility in an aqueous environment, which limits its use [20,21]. Therefore, to provide appropriate therapeutic care for pediatric patients, BU could be extemporaneously formulated as viscous oral suspensions [22]. The main limit of this therapy is the low residence time of the formulation on the esophageal mucosa that is not sufficient for the drug, which is suspended in a liquid vehicle, to solubilize and diffuse into the mucosa exerting its local anti-inflammatory action. This limitation is even more pronounced because of BU’s low water solubility [23,24,25]. To overcome this problem, a new ready-to-use mucoadhesive viscous liquid vehicle, named Fast Oral Solution Wagner (B1), has been used. Utilizing ready-to-use liquid vehicles is a valuable strategy for creating extemporaneous formulations. Since these vehicles are made with the highest quality ingredients, they guarantee the quality, safety, and efficacy required for medicines. In this manner, liquid bases provide a double advantage, reducing risks for pharmacists during compounding processes and expanding the range of formulation choices accessible to clinicians. B1 had the following qualitative composition: preserved water, sorbitol, carboxymethylcellulose sodium (CMC Na), hydroxypropyl-β-cyclodextrin (HP-β-CD), glycerol, potassium sorbate, citric acid, trisodium citrate dihydrate, and raspberry flavor. The aim of our work was to assess whether the presence of CMC Na could give mucoadhesive properties to the formulation and whether, by combining it with the solubilizing effects of cyclodextrins, it was possible to increase BU concentration on the mucosa. Thanks to the presence of HP-β-CD and CMC Na, it was possible to solubilize BU at a concentration of 0.7 mg/mL in the liquid base, obtaining stable oral solutions and increasing the residence time of the formulation on the esophageal mucosa, resulting in an evident improvement in the current therapeutic practice. 

## 2. Results and Discussion

The objective of this study was to design a liquid dosage form that could greatly improve the solubility of the API and guarantee a prolonged residence time on the esophageal mucosa for more effective local treatment of EoE in pediatric patients. For this reason, pharmaceutical excipients with peculiar characteristics were chosen. HP-β-CD was chosen for its safety in children and ability to improve the water solubility of lipophilic drugs such as glucocorticoids and, in particular, budesonide. CMC Na was chosen as a well-known viscosizing and mucoadhesive agent. Sorbitol was used in pediatric formulations for its sweetening and thickening abilities. Glycerol was used to facilitate the preparation of the formulation, acting as a humectant agent. Raspberry flavor was used to improve the palatability of the formulation. Preserved water and potassium sorbate were used as bases to obtain the aqueous vehicle and guarantee microbiological stability for a prolonged time. Finally, citric acid and trisodium citrate dihydrate were used to adjust the pH (around 5.7). An initial study was performed to highlight the mucoadhesive properties and the syringeability characteristics of the three liquid vehicles (B1, B2, and B3). Once these characteristics were defined, B1 and B3 were selected for further investigations since the presence of CMC Na conferred high mucoadhesive properties to the two liquid bases and their formulations. Rheological tests did not emphasize significant differences between the two liquid vehicles and their formulations. On the other hand, solubility and in vitro permeation studies showed that the presence of HP-β-CD allowed for a remarkable increase in BU solubility and permeability. After determining that the presence of the polymer and the cyclodextrin were necessary to achieve these relevant results, a comprehensive physical–chemical stability study demonstrated that thanks to B1, it was possible to obtain stable BU-based oral mucoadhesive solutions.

### 2.1. Mucoadhesive Property Determination

**Figure 1:**
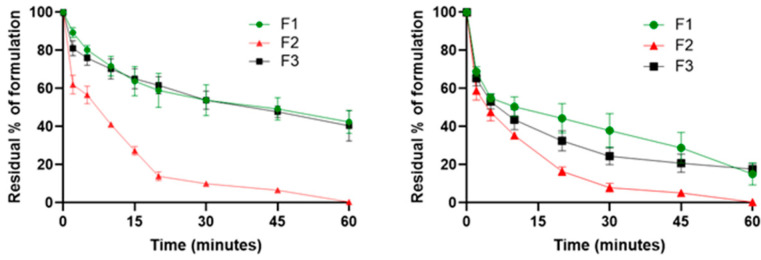
Mucoadhesive properties of F1 (BU-loaded mucoadhesive viscous oral solution), F2 (BU-loaded oral solution), and F3 (BU-loaded mucoadhesive viscous oral suspension) expressed as residual % of formulation on the esophageal mucosa as a function of time (minutes) at 25 °C (**left**) and 37 °C (**right**).

**Figure 2:**
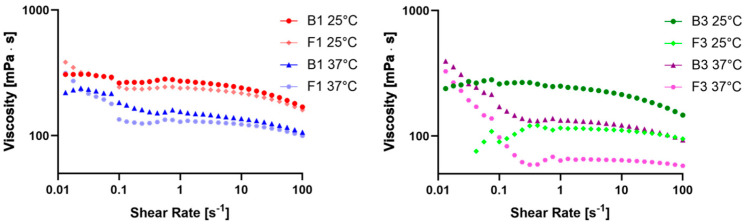
Plot of viscosity versus shear rates of B1 and F1 at 25 and 37 °C (**left**) and B3 and F3 at 25 and 37 °C (**right**).

The wash-off ex vivo experiment on freshly excised porcine mucosa helps to highlight the mucoadhesion of a dosage form on the first part of the esophagus and the flowability of the formulations. Several studies have been used to prove mucoadhesion by using the wash-off method and employing freshly excised porcine mucosa [16,26,27,28,29]. Ex vivo mucoadhesive studies on esophageal mucosa were performed on F1, F2, and F3 using Fluoresceine diacetate (FD) as a tracer. Results were expressed as the residual % of formulation at different time points. The residual % of formulation was calculated as the difference between the initial % of FD in the sample and the amount of FD registered from leakage after progressive washing with PBS. The results at 25 °C, as reported in Figure 1, showed that after 20 min from the experiment start, there was already a significant difference (*p* = 0.0003) between the CMC Na-containing formulations (F1 and F3) and F2, with a residual % of formulation adhered to the mucosa of about 60% and 14%, respectively. After 60 min, about 42% of F1 and 40% of F3 were still present on the esophageal mucosa, while F2 was completely washed off from the mucosa, and no substantial differences between the two samples were noticed (*p* = 0.7635). The experiment was also conducted at 37 °C to investigate the possible variation in the mucoadhesive properties of the formulations. At 37 °C, F1 and F3 showed a trend likely to 25 °C, but with an overall lower mucoadhesion. In fact, after 30 min since the start of the experiment, the residual percentage of the formulations adhered to the mucosa was around 40%, which decreased to 15% after 60 min. Regarding F2, no significant changes (*p* = 0.1366) were found compared to the experiment conducted at 25 °C. Current EoE therapies are based on oral viscous BU suspensions for topical treatment, and the choice to employ a viscous liquid vehicle for the administration of BU is justified by the more prolonged esophageal mucosa contact time obtained using a viscosizing agent. Starting from this knowledge, CMC Na has been chosen as viscosizing and mucoadhesive agent. The presence of CMC Na has already been demonstrated to be relevant for mucoadhesion, and this experiment confirmed that the presence of the polymer in both F1 and F3 granted significant mucoadhesive properties [30,31,32,33]. In this experiment, the mucoadhesion of the formulations was evaluated employing a piece of esophageal mucosa (3 × 2 cm). The 50% of the dosage forms (F1 and F3) that were washed away during the first 10 min of the experiment corresponds to the aliquot of the formulation that is not initially in contact with the mucosa. This outcome suggests that both F1 and F3 are able to flow freely and can spread through the entire length of the organ, adhering to the whole mucosal area of the esophagus. The temperature increase from 25 °C to 37 °C led to a reduction in mucoadhesion properties, and this could also be explained by the lowering of the viscosity of the formulations, as demonstrated with the rheological tests (Figure 2). Even though mucoadhesion decreased with higher temperatures, the reduced viscosity of the systems could be helpful to improve the spreadability of the mucoadhesive viscous liquids on the mucosa. Despite this, as expected, thanks to the formation of electrostatic interactions and hydrogen bonds between CMC and mucus at 37 °C, F1 and F3 still adhered to the mucosa after the initial 10 min for more than 60 min. Furthermore, the mucoadhesive study conducted at 37 °C was very helpful in mimicking physiological conditions, and the outcomes underlined that both the formulations containing CMC Na maintained their mucoadhesive properties. The low residence time of formulations on the esophageal mucosa was the main problem to overcome, and thanks to the presence of CMC Na, it was possible to create a highly mucoadhesive formulation for the local treatment of EoE.

### 2.2. Determination of Syringeability Properties

Since the current formulation used in hospitals (BU-based viscous suspension) is administered by using a syringe [4], to assess the possibility of handling and easy administration, a syringeability test was performed on the three formulations. The presence of CMC Na in the samples can affect their viscosity, which should be kept as low as possible at room temperature for easy administration and handling. Polymers dispersed in water can give a viscoelastic behavior to the liquid, which is the reason why the assay was performed by imparting exactly the same strength and acceleration, keeping only time as the testing determinant value [34,35].

The results shown in Table 1 highlight that F2 was the fastest to be completely eluted by using a syringe. The presence of CMC Na in both F1 and F3 led to a higher resistance to the imprinted force, but they still could be completely extruded by using a syringe, making them suitable for easy administration and handling.

### 2.3. Solubility Study

BU solubility was investigated by using the shake flask method. Based on the outcomes from mucoadhesion and syringeability studies, the B2 liquid base resulted in low mucoadhesive properties, which is the reason why further studies were conducted on B1 and B3. Solubility studies were performed in triplicate in B1 and B3 liquid bases and in water (used as reference standard). The analyses highlighted a remarkable increase in solubility in B1. In particular, BU water solubility was 0.019 mg/mL ± 0.003 mg/mL (in accordance with the literature [36]) and 0.033 ± 0.007 mg/mL in B3, while in B1, it was 0.737 mg/mL ± 0.020 mg/mL with an increase between 22 and 39 times. An increase in BU solubility was observed in both B1 and B3 compared with water. B1 resulted in having the highest solubility increase, and the reasons for such results can be found in the composition of the liquid vehicles. HP-β-CD can form water-soluble inclusion complexes that can enhance the apparent water solubility of lipophilic drugs since it is characterized by a hydrophilic external structure and a lipophilic core, which can include entire drugs or parts of them. Such outcomes are confirmed by a previous study conducted in 2019 by Laquintana et al. on budesonide and HP-β-CD [36]. The remarkable increase in BU water solubility is attributed to the presence of HP-β-CD and also to the presence of CMC Na. In fact, the B1 formula includes the presence of CMC Na, which, in combination with cyclodextrins, can further improve complexation ability, forming a ternary complex, and apparent aqueous solubility of the drugs, as demonstrated by many studies published in the scientific literature [21,37,38,39]. Finally, the presence of glycerol as a co-solvent could also increase BU solubility in B1. On the other hand, a slight solubility increase compared with water was also highlighted in B3 because of the presence of both CMC Na, which was proven to increase the aqueous solubility of various drugs thanks to the formation of hydrogen bonds between the polymer and the API, and glycerol for its co-solvency properties. In this case, the drug solubility increase observed in B1 allowed for the preparation of liquid BU solutions with dosages that cover both pediatric and adult patient therapies. Moreover, because of the solubilization of the API, it is possible to bypass the dissolution step of the drug, typical of suspended systems, allowing for an increase in its local effect.

### 2.4. Evaluation of Rheological Properties

The evaluation of rheological properties was performed on the B1 and F1 and B3 and F3 samples. First, the viscosity behavior of the samples was evaluated by correlating the viscosity (mPa·s) to the shear rate (s^−1^). The viscosity curves (Figure 2) are plotted over a logarithmic scale in a shear rate range between 0.1 s^−1^ and 100 s^−1^. Some significant data regarding the change in viscosity at several high shear rates are reported in Table 2, as well as the percentual variation in viscosity between 56.3 and 100 s^−1^ (Δ viscosity 56.3 vs. 100 s^−1^ (%)). Moreover, the regression coefficients of the four samples at both 25 and 37 °C were calculated and reported in Table 3.

The evaluation of the viscosity at different shear rates and temperatures indicated that all samples had very similar behavior, except for F3, and they were non-Newtonian pseudoplastic fluids. Furthermore, the increase in temperature from 25 °C to 37 °C led to a viscosity reduction, as in the case of most of the liquids, facilitating the swallowability of patients with EoE [40,41,42]. All samples had a flow behavior index lower than 1, confirming that they were non-Newtonian pseudoplastic fluids with a high yield value, except for F3 at 25 °C. This behavior was also confirmed by the Δ viscosity trend shown in Table 2 and also by studies on CMC Na solutions conducted by Ghannam and Esmail and Edali et al. [43,44].

**Figure 3:**
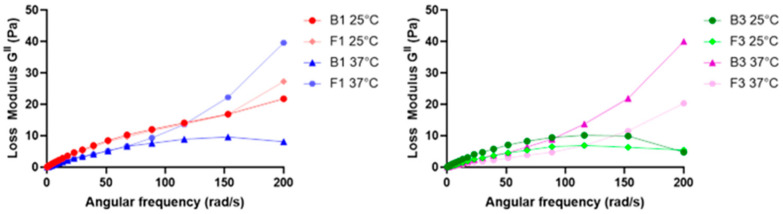
Plot of G^II^ versus ω of B1 and F1 at 25 and 37 °C (**left**) and B3 and F3 at 25 and 37 °C (**right**).

To determine the loss factor (tan δ), and thus the elastic or viscous behavior of B1, B3, F1, and F3, dynamic shear measurements made in an angular speed range between 0.676 and 67.7 (rad/s) at 25 °C and 37 °C were considered [16]. A plot of the loss modulus (G^II^) against the angular speed (ω) of all tested samples is shown in Figure 3.

The loss factor (tan δ) could be calculated for all samples, and the mean values and their respective standard deviations (SDs) are reported in Table 2. G^II^ and, consequently, tan δ were calculated to obtain further information regarding the viscous or elastic behavior of our tested samples. In fact, when this value is smaller than the unit, a sample is more elastic than viscous, and vice versa [16,45]. Since in all the tested samples, the loss factor was found to be higher than the unit, it was considered extra data that confirmed the formulations were liquids characterized by high viscosity. Since the higher the viscosity the higher the residence time, thanks to the presence of CMC Na, it was possible to increase system viscosity, and this confirmed the results shown above regarding the mucoadhesion and residence time of F1 and F3.

### 2.5. In Vitro Diffusion–Permeation Study

**Figure 4:**
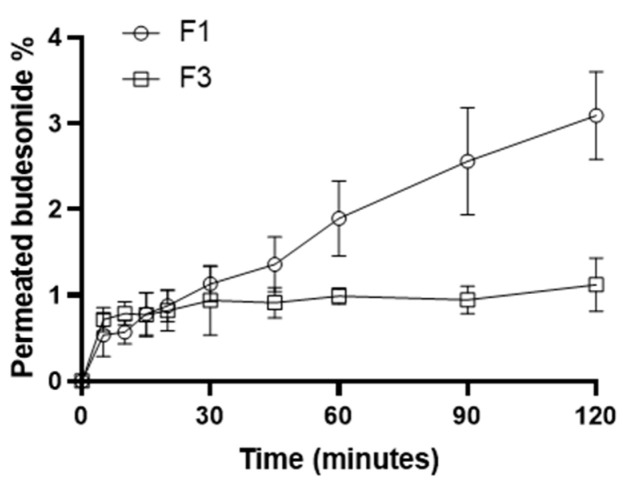
Permeation profiles of the F1 and F3 formulations.

An in vitro diffusion–permeation study, using a hydrophilic membrane, was performed to evaluate the kinetic properties of F1, comparing them to those of the suspended formulation (F3), which was used as a control standard. Cellulose acetate (with a cutoff ranging from 12 to 14 kDa), considered a low flux membrane, is a hydrophilic membrane that was used in several diffusion and permeation studies of lipophilic drugs [46,47]. The experiment, conducted in 120 min, allowed for defining the BU permeation profiles for both tested formulations (Figure 4).

The permeated BU amounts allowed us to calculate the fluxes (J), the Apparent Permeability coefficients (P_app_), and the Transport Enhancement Ratio (R) of the formulations under investigation (Table 4) [48]. F1 showed higher P_app_ and J than F3, resulting in an R of about 2.7. BU resulted in permeating more if solubilized and not if suspended, indicating that solubilization allowed for skipping the dissolution process of the API in the liquid vehicle and the drug to diffuse directly across the membrane. Moreover, it has long been demonstrated that CDs can be used as penetration enhancers because they can deliver the carried drug into lipophilic environments such as the esophageal mucosa. BU was released by the CD and permeated directly, requiring a shorter time than the control suspension [48,49,50]. A higher J leads to an increase in drug permeation, resulting in a higher BU concentration in the treated area. This peculiarity gives an evident advantage, especially in the case of the local treatment of pathological conditions of the esophageal mucosa, where the low residence time of the formulation limits the accumulation of the drug and thus the effectiveness of the treatment [51]. This evidence led us to select B1 as the most promising vehicle to deliver BU for the treatment of EoE, thanks to the enhanced water solubility and mucoadhesive properties. 

### 2.6. Physical–Chemical Stability Study

**Figure 5:**
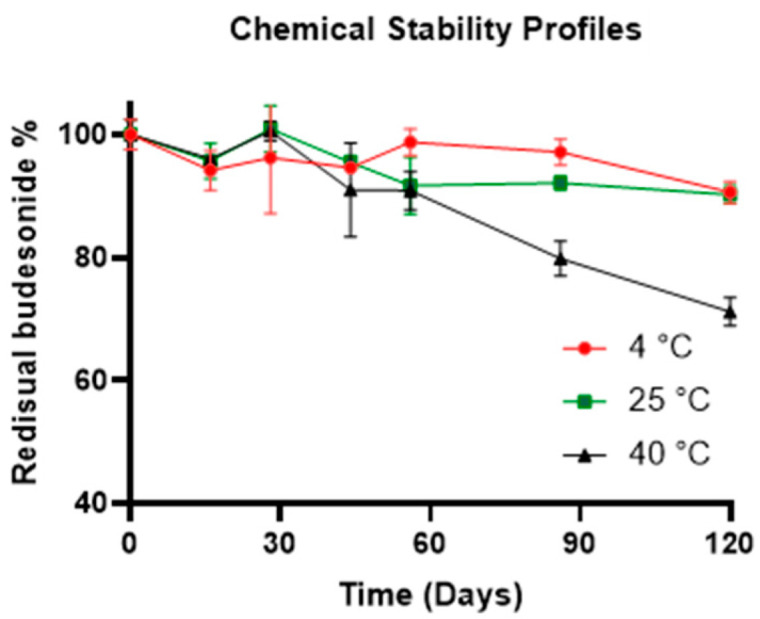
Chemical stability profiles of the F1 formulations at different storage conditions.

For the physical–chemical stability study, samples of F1 were prepared. The latter were subjected to three different storage conditions as follows: 4 °C in a refrigerator, 25 °C in a thermostat chamber, and 40 °C and 60% relative humidity (RH) in a climatic chamber (Climacell; MMM Medcenter, Munico, Germany). Over 120 days, several HPLC analyses conducted on the samples demonstrated that the API was stable if stored at 4 °C or 25 °C with a residual drug content % of 90.61 ± 1.80 (0.640 mg/mL ± 0.012 mg/mL) and 90.26 ± 1.47 (0.638 mg/mL ± 0.010 mg/mL), respectively (Figure 5). The HPLC method was developed to evaluate the residual content of BU in the formulations analyzed at different time points, and it was demonstrated to be stability-indicating. BU and F1 subjected to forced stress tests were analyzed, and the HPLC chromatograms showed that degradants of both the excipients of the liquid base and the drug had different retention times than the API (Figure S2A–F). Chemical stability analyses did not highlight the presence of any degradants of BU in F1 stored at either 4 °C or 25 °C (Figure S2B). On the other hand, the formulation stored at 40 °C showed a dramatic decrease in residual API content, demonstrating a shelf life shorter than 60 days.

Moreover, during the whole stability study, no change in color or the presence of a precipitate was noticed, and the pH values of the formulation were found to be stable (pH 5.75) for the formulations at all the storage conditions. This study demonstrated that BU-based liquid formulations, realized using B1, had high drug stability and a formulation shelf life longer than 120 days at common storage conditions (4 °C and 25 °C).

## 3. Materials and Methods

### 3.1. Materials

BU, highly purified grade water, Nipagin, Nipasol, CMC Na, HPβCD, sorbitol, vegetable glycerol 85% Ph-EUR-E422, potassium sorbate, citric acid, and Fast Oral Solution Wagner were kindly gifted by “Farmalabor srl.” (Canosa di Puglia, Italy). Trisodium citrate trihydrate was purchased from the “Carlo Erba” company (Cornaredo, Italy). HPLC-grade methanol (MeOH), HPLC-grade water, and trifluoroacetic Acid (TFA) (Lot: L0130), were purchased from “Levanchimica” (Bari, Italy) and were used for sample preparation and HPLC analysis.

### 3.2. Preparation of BU Formulations

Two different liquid vehicles (B2 and B3) were prepared to carry out a comparative study with B1 for syringeability and mucoadhesive properties and also for the in vitro permeation study. Both B2 and B3 had the same composition as B1, except for CMC Na, which was missing in B2, and HP-β-CD, which was substituted by sorbitol in the same amount in B3 (Table 5). BU was then solubilized/dispersed in the three liquid vehicles to obtain a final concentration of 0.7 mg/mL (the formulations composed of the liquid vehicles containing BU at a concentration of 0.7 mg/mL are indicated in the text as F1, F2, and F3, respectively).

### 3.3. HPLC Method and Calibration Curve

The analyses were carried out by using an Agilent 1260 Infinity quaternary LC VL system equipped with a variable wavelength detector and the software OpenLab CDS (Version C.01.06 Agilent Technologies, Santa Clara, CA, USA). A Zorbax Eclipse plus C18 150 mm × 4.6 mm, 5 µm column (Agilent Technologies, Santa Clara, CA, USA) was used. The mobile phase was composed of 70% *v*/*v* methanol and 30% *v*/*v* water, containing 0.1% *v*/*v* of trifluoracetic acid (TFA). The flow rate and the temperature were set at 1.0 mL/min and 35 °C, respectively, and ultraviolet detection was carried out at 254 nm. The retention time of the drug was about 5.8 min, and the volume of each injected sample was 20 µL (Figure S1). A calibration curve was realized by solubilizing 5 mg of BU with 5 mL of methanol: water 70:30 *v*/*v* in a volumetric flask. Consecutive dilutions were performed, and the linearity of the HPLC method was demonstrated in a concentration range between 1000 µg/mL and 1 µg/mL (*R^2^* > 0.9996).

### 3.4. Mucoadhesive Properties Determination

To determine the mucoadhesive properties of the tested formulations, in vitro mucoadhesive studies were performed on freshly excised bovine esophagus with a slightly modified wash-off method [51]. The three formulations (F1, F2, and F3) loaded with 1 mg/mL of fluorescein diacetate (FD) were tested. The formulations were applied to 3 × 2 cm pieces of esophagus set on a 45° angle inclined support at room temperature (25 ± 0.5 °C) and 37 ± 0.5 °C. Then, 60 mL of phosphate buffer solution (PBS pH 6.8, 100 mM) flowing down the mucosa at 1.0 mL/min was collected in a glass beaker, in which the collected liquid was agitated under magnetic stirring to make the sample homogeneous. Samples were collected at different time points. All collected samples were shaken for 30 s, and to hydrolyze FD to sodium fluorescein, 5M NaOH was added. These solutions were incubated at 37 °C for 20 min. The samples were centrifugated, and an aliquot of the supernatant of each sample was used to evaluate the fluorescence intensity using a Victor X3 (PerkinElmer, Waltham, MA, USA). Mucoadhesive properties (%) were calculated by quantifying the residual FD on the esophageal mucosa using the following formula:

(1) $$Mucoadhesion\left(\%\right)=100-\left(\frac{Fluorescence\left(sample\right)}{Fluorescence\left(reference\right)}\times 100\right)$$

### 3.5. Determination of Syringeability Properties

The syringeability properties of the three formulations (liquid vehicles loaded with BU at a concentration of 0.7 mg/mL) were evaluated using a modification of the piston-syringe method by Schuetz et al., 2008 [35,52]. The purpose of the evaluation was to determine the handling properties and feasibility of administering the formulation with a syringe. Testing was performed by placing a 1 kg weight on a syringe with a 21 Gauge diameter nozzle. The syringe was fixed to a vertical support using a clamp. The syringe was filled with 18 mL of the formulation and kept at 25 °C, and the force, corresponding to 9.8 N, was kept constant during the experiment. The time required to completely empty the syringe was measured five times for each sample.

### 3.6. Solubility Study

A solubility study was performed to evaluate the intrinsic solubility (S_0_) of BU in water, B1, and B3 at 25 °C. In particular, the saturation shake-flask method was used [53]. Triplicate samples were performed, putting an excess of the drug in stoppered glass vials containing water or B1 or B3. They were sonicated for 10 min using an Ultrasonic Cleaner CWB02 (FIOA International srl., Arezzo, Italy) and kept under magnetic stirring for 72 h at 25 °C to achieve solubility equilibrium. After that, to separate the drug excess, aliquots of the samples were put in 2.0 mL polypropylene microcentrifuge tubes and centrifuged at 13,200 rpm for 10 min at 25 °C. Aliquots of 0.5 mL of the supernatant were diluted in methanol into a 25 mL volumetric flask and then analyzed in triplicate using the above-described HPLC method. Since the polymer (CMC Na) precipitated because of its low solubility in methanol, before being analyzed, B1 and B3 samples were vigorously mixed and put in a bath sonicator for 2 min (to extract the drug from the precipitated polymeric matrix) and centrifuged for 10 min at 25 °C and 13,200 rpm.

### 3.7. Evaluation of Rheological Properties

A HAAKE^TM^ MARS iQ rheometer (Thermo Fisher Scientific, Waltham, MA, USA) equipped with an aluminum plate–plate geometry (25 mm diameter, 0.5 mm gap between the plates) was used to obtain viscosity curves. The experiment was conducted at a temperature of 25 °C and 37 °C on four different samples including B1, F1, B3, and F3. The Δ shear stress as a function of Δ shear rate was measured over a range of 0.1 to 100.0 s^−1^ for the pharmaceutical vehicles (B1 and B3) and pharmaceutical formulations containing a BU concentration of 0.7 mg/mL (F1 and F3). The data curve fitting was performed using GraphPad Prism 9.3 (Boston, MA, USA) following the Ostwald-de Waele Equation (2) with a weighted sum of squares (1Y2):

(2) $$\tau =K\;{ɣ˙}^{n}\;$$

where *τ* (Pa) is the shear stress, ɣ˙ (s^−1^) is the shear rate, K (Pa s*^n^*) is the consistency index, and *n* is the flow behavior index. The Δ viscous modulus of the pharmaceutical vehicles (B1 and B3) and pharmaceutical formulation with a BU concentration of 0.7 mg/mL (F1 and F3) as a function of the change in angular velocity (ω) over a range of 0.3 to 200 rad/s was evaluated. The obtained data from storage modulus (G′) and G″ were used to calculate tan *δ* as the average ratio and standard deviation of G″/G′ in a ω range between 0.676 and 67.7 rad/s.

### 3.8. In Vitro Diffusion–Permeation Study

In vitro permeation studies were conducted using a Franz diffusion cell. The apparatus consisted of a donor compartment, where 0.5 mL of the tested formulations (F1 and F3) was placed, and a receptor compartment containing 9.5 mL of a 10% *w*/*v* HPβCD solution in phosphate buffer (PBS) with a pH of approximately 6.8. The presence of cyclodextrin in the receptor solution increased the solubility of BU. The two compartments were separated by an artificial cellulose acetate membrane with a cutoff ranging from 12 to 14 kDa. The apparatus was kept in a water bath at a controlled temperature of 37 °C, and multiple withdrawals of 250 µL were taken at different time points over a period of 2 h. At the end of the experiment, a 100 µL sample was withdrawn from the donor compartment to measure the residual drug quantity. All samples were analyzed in triplicate using the HPLC method described in Section 3.3. Results were processed to calculate J as the slope of the linear plot of the drug amount in the receptor compartment (Q) against time and *P_app_* using the following equation:

(3) $$J=\frac{dQ}{\left(dt\times A\right)}=P_{app}\times C_{d}$$

*R* was also calculated as the ratio of F1 *P_app_* and F3 *P_app_*_._

### 3.9. Physical–Chemical Stability Study

A comprehensive physical–chemical stability study was conducted on the formulation of F1. Samples at a concentration of 0.7 mg/mL were prepared and stored in 60 mL amber containers away from light exposure at three different storage conditions as follows: 4 °C in a refrigerator, 25 °C in a thermostat chamber, and 40 °C and 60% RH in a climatic chamber (Climacell; MMM Medcenter, Munico, Germany). During a 120-day period, the chemical stability of BU in the samples was evaluated by the HPLC method, quantifying the residual drug content and the eventual presence of degradants at several time points (0, 16, 28, 44, 56, 86, and 120 days). On the other hand, to confirm the physical stability of the formulation, its appearance and pH values were monitored.

### 3.10. Statistical Analysis

The results are expressed as the mean ± SD from independent experiments. For ex vivo mucoadhesive studies on esophageal mucosa, statistical significance was calculated using a two-way analysis of variance (ANOVA) test (GraphPad Prism version 9.3). Differences were considered significant at the *p* < 0.05 level.

## 4. Conclusions

The formulation F1 presented in this paper is a mucoadhesive viscous oral solution that has peculiar characteristics, resulting in a tangible advancement in EoE treatment. Thanks to the presence of HP-β-CD, B1 is a novel liquid vehicle that allows for the preparation of stable BU mucoadhesive viscous solutions at a concentration of up to 0.7 mg/mL, resulting in its suitability for all types of patients, in particular, pediatric patients. Moreover, since BU is already solubilized in the liquid vehicle, the dissolution step is bypassed, guaranteeing an immediate diffusion and permeation of the API, as demonstrated by the comparative in vitro diffusion–permeation study. Furthermore, the mucoadhesion and rheological tests highlighted that CMC Na confers pronounced mucoadhesive properties to the formulation, guaranteeing a high residence time on the esophageal mucosa. In this way, the mucoadhesive solution can be effective even at low dosages and requires fewer administrations per day than the viscous oral suspensions currently used for EoE therapy. These properties could result in an enormous step forward in the search for a more effective and safe local treatment of EoE compared with the currently available therapeutic options.

## 5. Patents

An Italian pending patent was submitted to the Italian Office for Patent and Brands for the industrial production of oral mucoadhesive vehicle B1 and BU oral mucoadhesive solution (pending patent number 102023000021669, 20 October 2023).

## Figures and Tables

**Table 1:** Syringeability properties at 25 °C of the formulations.

Formulation	Syringeability * (s)
F1	3.310 ± 0.266
F2	2.014 ± 0.210
F3	4.022 ± 0.318

* Syringeability is expressed as the time (s) required for complete elution of 18 mL of the preparation at 25 °C. The result is expressed as the mean of five different experiments ± its relative standard deviation (S.D.).

**Table 2:** Viscosity at different shear rate values, Δ viscosity 56.3 vs. 100 s^−1^ (%), and loss factor values of the samples.

Sample	Temperature (°C)	Viscosity (mPa·s)			Δ Viscosity 56.3 vs. 100 s^−1^ (%)	Loss Factor (tan δ)
		56.3 (s^−1^)	75 (s^−1^)	100 (s^−1^)		
B1	25	190.65	180.31	169.23	−11.23	9.68 ± 6.28
B1	37	116.36	111.37	106.43	−8.534	4.33 ± 2.43
F1	25	178.91	169.88	160.40	−10.35	12.73 ± 9.98
F1	37	107.87	103.81	99.766	−7.513	24.49 ± 22.49
B3	25	166.77	156.83	146.85	−11.94	4.90 ± 3.69
B3	37	102.38	98.083	93.071	−9.092	17.15 ± 19.88
F3	25	100.78	97.81	94.775	−5.959	15.81 ± 12.68
F3	37	60.167	59.075	57.887	−3.789	32.99 ± 30.62

**Table 3:** Regression coefficients of the various samples.

Sample	Temperature (°C)	*K* (Pa s*^n^*)	*n*	*R* ^2^
B1	25	0.2553	0.9517	0.9935
B1	37	0.1598	0.9178	0.9949
F1	25	0.2438	0.9239	0.9872
F1	37	0.1439	0.9097	0.9653
B3	25	0.1779	0.9490	0.9933
B3	37	0.1200	0.8716	0.9554
F3	25	0.1008	1.0360	0.9647
F3	37	0.0835	0.8758	0.8913

**Table 4:** In vitro permeation parameters of F1 and F3 formulations.

Formulation	J [µg h^−1^ cm^−2^]	P_app_ (×10^−6^) [cm s^−1^]	R
F1	8.05	3.19	2.72
F3	2.96	1.18	

**Table 5:** Qualitative composition of B1, B2, and B3.

Qualitative Composition of the Liquid Vehicles		
B1	B2	B3
CMC Na	/	CMC Na
HP-β-CD	HP-β-CD	/
Sorbitol	Sorbitol	Sorbitol
Glycerol	Glycerol	Glycerol
Potassium sorbate	Potassium sorbate	Potassium sorbate
Citric acid	Citric acid	Citric acid
Trisodium citrate dihydrate	Trisodium citrate dihydrate	Trisodium citrate dihydrate
Raspberry flavor	Raspberry flavor	Raspberry flavor
Preserved water	Preserved water	Preserved water

## Data Availability

Data are contained within the article.

## Supplementary Materials

The following supporting information can be downloaded at: https://www.mdpi.com/article/10.3390/ph17050550/s1, Figure S1: Representative chromatogram of the BU analytical standard solubilized in methanol.; Figure S2: Representative chromatograms of B1 (A), F1 after 120 days stored at 25 °C (B), BU subjected to forced stress test in presence of HCl 1N for 24 h (C), BU subjected to forced stress test in presence of NaOH 1N for 24 h (D), F1 subjected to forced stress test in presence of HCl 1N for 24 h (E), and F1 subjected to forced stress test in presence of NaOH 1N for 24 h (F).
