# Supplementary material for: Mucoadhesive Budesonide Solution for the Treatment of Pediatric Eosinophilic Esophagitis
Source: Pharmaceuticals (Basel). 2024 Apr 24;17(5):550. doi: 10.3390/ph17050550 (PMC11124118; doi:10.3390/ph17050550)
Supplement: Supplementary file 1 [file pharmaceuticals-17-00550-s001.zip › pharmaceuticals-2957000-supplementary.pdf]

Supplementary Materials for

Mucoadhesive Budesonide Solution for the Treatment of Pediatric Eosinophilic Esophagitis

Antonio Spennacchio [1], Antonio Lopalco [1], Giuseppe Francesco Racaniello [1], Annalisa Cutrignelli [1], Flavia Maria la Forgia [2], Sergio Fontana [2], Fernanda Cristofori [3], Ruggiero Francavilla [3], Angela Assunta Lopedota [1] and Nunzio Denora [1,*]

1 Department of Pharmacy-Pharmaceutical Sciences, University of Bari Aldo Moro, 70125 Bari, Italy
2 Centro Studi e Ricerche "Dr. S. Fontana 1900–1982", Farmalabor s.r.l., 76012 Canosa di Puglia, Italy
3 Interdisciplinary Department of Medicine, Paediatric Section, University of Bari Aldo Moro, Paediatric Hospital Giovanni XXIII, 70125 Bari, Italy
* Correspondence: nunzio.denora@uniba.it; Tel.: +39-080-5442767

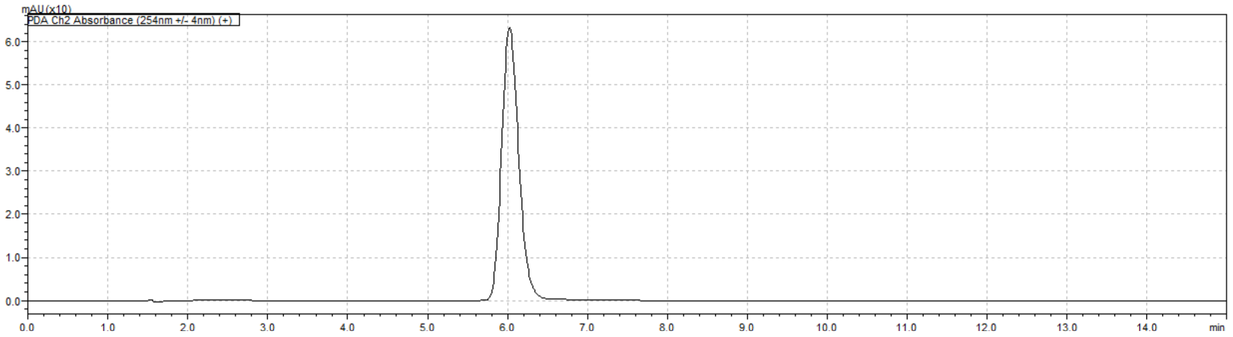

Figure S1. Representative chromatogram of BU analytical standard solubilized in methanol.

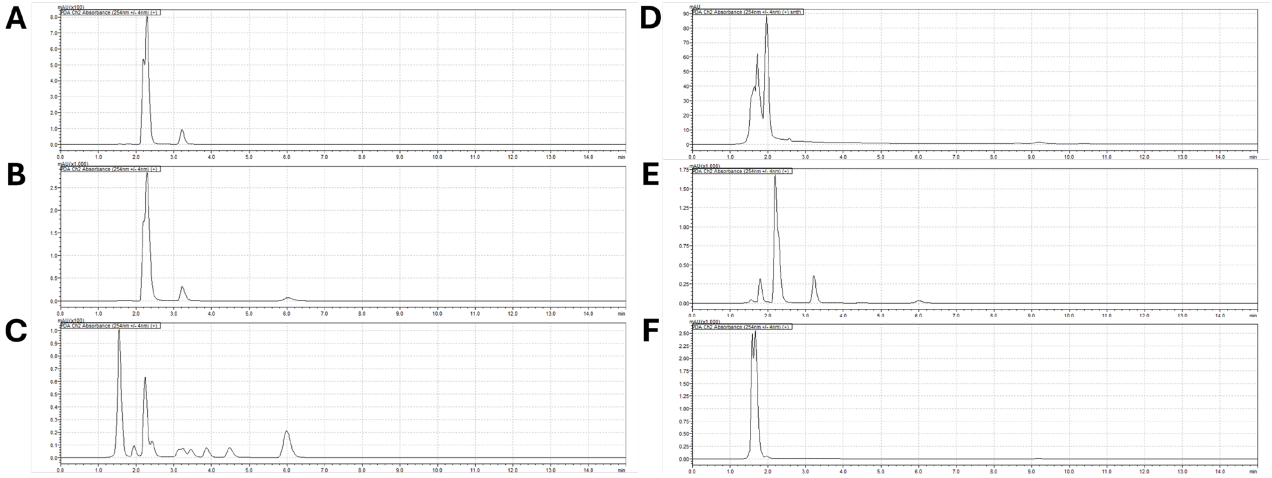

Figure S2. Representative chromatograms of B1 (A), F1 after 120 days stored at 25 °C (B), BU subjected to forced stress test in presence of HCl 1N for 24 hours (C), BU subjected to forced stress test in presence of NaOH 1N for 24 hours (D), F1 subjected to forced stress test in presence of HCl 1N for 24 hours (E), F1 subjected to forced stress test in presence of NaOH 1N for 24 hours (F).
